# Deep data analysis via physically constrained linear unmixing: universal framework, domain examples, and a community-wide platform

**DOI:** 10.1186/s40679-018-0055-8

**Published:** 2018-04-30

**Authors:** R. Kannan, A. V. Ievlev, N. Laanait, M. A. Ziatdinov, R. K. Vasudevan, S. Jesse, S. V. Kalinin

**Affiliations:** 1 The Institute for Functional Imaging of Materials, Oak Ridge National Laboratory, Oak Ridge, TN 37831 USA; 2 Computer Science and Mathematics Division, Oak Ridge National Laboratory, Oak Ridge, TN 37831 USA; 3 The Center for Nanophase Materials Sciences, Oak Ridge National Laboratory, Oak Ridge, TN 37831 USA

**Keywords:** Unmixing, Image segmentation, Scanning probe microscopy, Matrix factorization, Big data, High performance

## Abstract

Many spectral responses in materials science, physics, and chemistry experiments can be characterized as resulting from the superposition of a number of more basic individual spectra. In this context, unmixing is defined as the problem of determining the individual spectra, given measurements of multiple spectra that are spatially resolved across samples, as well as the determination of the corresponding abundance maps indicating the local weighting of each individual spectrum. Matrix factorization is a popular linear unmixing technique that considers that the mixture model between the individual spectra and the spatial maps is linear. Here, we present a tutorial paper targeted at domain scientists to introduce linear unmixing techniques, to facilitate greater understanding of spectroscopic imaging data. We detail a matrix factorization framework that can incorporate different domain information through various parameters of the matrix factorization method. We demonstrate many domain-specific examples to explain the expressivity of the matrix factorization framework and show how the appropriate use of domain-specific constraints such as non-negativity and sum-to-one abundance result in physically meaningful spectral decompositions that are more readily interpretable. Our aim is not only to explain the off-the-shelf available tools, but to add additional constraints when ready-made algorithms are unavailable for the task. All examples use the scalable open source implementation from https://github.com/ramkikannan/nmflibrary that can run from small laptops to supercomputers, creating a user-wide platform for rapid dissemination and adoption across scientific disciplines.

## Introduction

The development of physical and spectroscopic imaging methods in the last two decades has given rise to large multidimensional datasets, with examples including electron energy loss spectroscopy imaging in (scanning) transmission electron microscopy [1–4], bias and time spectroscopies in scanning probe microscopy [5–8], hyperspectral Raman and optical imaging [9–12], and spatially resolved mass spectrometry measurements [13–15].

In many of these techniques, the measured signal can be (with good approximation) presented as a linear combination of spectra, i.e.,

1 $$S\left( {{\mathbf{x}}, {\mathbf{R}}} \right) = \sum\limits_{i = 1}^{k} {a_{i} \left( {\mathbf{x}} \right)w_{i} \left( {\mathbf{R}} \right) + N},$$

where **x** is the spatial variable, **x** = (*x*,*y*), **R** is the vector parameter variable, $w_{i} \left( {\mathbf{R}} \right)$ is the individual spectra (sometimes called ‘endmembers,’ ‘factors,’ or ‘components’), and *a*_*i*_(**x**) are corresponding spatial maps (also called abundance maps) and *N* defines the noise (not considered here). For example, *w*_*i*_(**R**) can be optical spectra in Raman and hyperspectral imaging, mass spectra, energy loss spectra in electron microscopy, force–distance curves in atomic force microscopy, etc. The loading maps *a*_*i*_(**x**) correspond then to local weightings of each spectrum, with examples such as concentration of relevant chemical species, phases, etc.

A special case of linear mixing is the linear imaging technique, for which the measured image $I(\varvec{x})$, is given by the convolution of an *ideal image* (representing material properties) $I_{0} \left( {\varvec{x} - \varvec{y}} \right)$ with the resolution function dependent on probe geometry, $F(\varvec{y})$:

2 $$I(\varvec{x}) = \int {I_{0} \left( {\varvec{x} - \varvec{y}} \right)F(\varvec{y})d\varvec{y} + N(\varvec{x})},$$

where $N(\varvec{x})$ is the noise function. While in general the linearity of particular imaging mode needs to be proven, it is considered to be a reasonable approximation in the case of many optical [16], mass spectrometry [17], scanning probe [18–21], and electron microscopy techniques [22]. The important aspect of Eq. (2) is that finite spatial resolution does not affect the linearity of the mixture, making analysis via Eq. (1) universal.

In certain cases, the elementary contributions *w*_*i*_(**R**) in Eq. (1) are known, for example from tabulated data for the specific system. In this case, the problem is reduced to the determination of the unknown weight coefficients *a*_*i*_(**x**) via minimal least square regression. Since least squares is a convex optimization, there exists a unique *a*_*i*_(**x**) given *w*_*i*_(**R**) [23]. At other times, it is necessary to solve a constrained least squares [23, 24] problem, such as non-negativity [25], box [26, 27], etc. But in all cases the separation of spectrum into a linear combination of known components with unknown coefficients presents a relatively straightforward problem.

However, in many cases the functional form of the endmembers is unknown, leading to a paradoxical problem where we need to determine both loading maps $a_{i} \left( {\mathbf{x}} \right)$ and endmember spectra *w*_*i*_(**R**) from multiple realizations of the experimental observations *S*(**x**,**R**). This constitutes the classical linear unmixing problem [28, 29].

The classical tool to address it is principal component analysis (PCA), known since work by Pearson [30] in the early twentieth century. PCA has started to become popular with the increase of the data size, e.g., from internet applications [31], as a first step of exploratory data analysis for visualizing high dimensional data. Multiple applications of PCA for hyperspectral optical imaging [32], EELS [33–36], mass spectrometry [37, 38], and scanning probe microscopy [39–42] have been further reported. However, while it is an extremely powerful exploratory data analysis tool, and is well defined from the information theory perspective, PCA-derived components lack physical constraints. For example, PCA components of the (positively defined) EELS signal will have negative regions, automatically precluding physical interpretation. This consideration highlights the (to-date) limited applicability of linear unmixing techniques in physical imaging.

However, developments in matrix factorization have enabled a considerably broader spectrum of linear unmixing techniques that allow superimposing a large number of constraints on either loading maps or endmembers. It can be argued that in cases when the statistically imposed constraints match the anticipated physics of the system, the unmixing will directly provide the insight to the latter.

In this manuscript, we present a review of matrix factorization (MF) approaches, as well as a tutorial for domain experts on how these new approaches can be applied to a variety of imaging modalities. We discuss the different physical constraints that can be placed on the endmembers and the spatial maps, that can result in more physical meaningful results, and show test cases with examples ranging from spatially resolved mass spectrometry, to electron microscopy, scanning tunneling, and X-ray microscopy. An overview of matrix factorization is provided in “Notations” section. Constraints are discussed in “Matrix factorization” section, and examples of hyperspectral imaging and MF-based images analysis are presented in “Matrix factorization framework (MFF)” and “Domain specific applications” sections.

## Notations

We begin with introducing the conventions used in the equations. We use capital case letter such as *A* to denote matrices and lower case *a* for vectors. The one indexed lower case such as *a*_*i*_ is a scalar value and represents the vector element at ‘*i*.’ Similarly, the two-indexed upper/lower cases such as *A*_*ij*_ or *a*_*ij*_ represents the scalar value—also called element of the matrix at the location (*i*,*j*). We often require a scalar value for the entire matrix or vector, and one example that can be computed is the so-called matrix or vector norm. More formally a norm is represented as $|\left| A \right||_{q} :A \in {\mathcal{R}}^{m \times n} \to {\mathcal{R}}$. The typical values for *q* are 1, 2, and *F* called as ℓ1-norm, ℓ2-norm, and Frobenius norm, respectively. Table 1 defines each of these norms, and also offers a quick reference for many of the terms used in this paper. Also, if there is a comparison relation defined between a matrix/vector and a scalar, the relations are defined against every element in the matrix or a vector to the vector. For e.g., *A* > 0 means every element in the matrix is non-negative and similarly for a vector it is represented as *a* > 0.

**Table 1:** Notations

Notation	Remarks
$A \in {\mathcal{R}}^{m \times n}$	Capital case letter generally denotes a matrix of size *m* × *n*
$a \in {\mathcal{R}}^{m}$	Lower case letter denotes a column vector of length *m*
*A*_*ij*_ or *a*_*i*_	A scalar/element from the matrix at location (*i*,*j*) or a vector element at *i*
||*A*||_*F*_	$$\sqrt {\mathop \sum \nolimits_{i = 1}^{m} \mathop \sum \nolimits_{j = 1}^{n} A_{ij}^{2} }$$—square root of the sum of the squares of all the elements of the matrix
||*A*||_1_	$$\sum\nolimits_{i = 1}^{m} {\sum\nolimits_{j = 1}^{n} {\left| {A_{ij} } \right|} }$$—sum of absolute values of all the elements. Here absolute value means the non-negative value without its sign
||*a*||_2_	$$\sqrt {\sum\nolimits_{i = 1}^{m} {a_{i}^{2} } }$$—square root of the sum of the squares of all the elements of the vector
*μ*	Mean of a vector
*KL*(*P*||*Q*)	Defines the similarity between two matrices *P* and $Q$ as $\sum\nolimits_{i = 1}^{m} {\sum\nolimits_{j = 1}^{n} {\left( {P_{ij} \log \frac{{P_{ij} }}{{Q_{ij} }} } \right)} }$

## Matrix factorization

In this section, we will introduce the matrix factorization problem and its connection with the linear unmixing explained above. Subsequently, we explain our matrix factorization framework (MFF) that offers a pragmatic framework of incorporating many real-world physical constraints. We introduce the popular linear unmixing techniques principal component analysis (PCA) and non-negative matrix factorization (NMF) under this framework and finally, discuss the examples of the two real-world constraints, sparsity and spatial smoothness, as preferential soft constraints with non-negativity on endmembers. The aim of this section, is to provide domain scientists sufficient information to extend the existing off-the-shelf algorithms with additional domain constraints they will encounter during their experiments, hopefully facilitating better understanding and use of multidimensional spectral data.

Matrix factorization is the problem of decomposing the input matrix into two or more matrices—called factors, such that the product of these factors is close to the input matrix. Typically, the rank of these factors will be much less than the rank of the input matrix and is termed as a “low rank approximation” in numerical computing. The rank is similar to number of principal components in PCA. However, in the Big Data literature [24, 43], as opposed to low-rank approximation, the community liberally calls this problem a “matrix factorization” as it determines the factors for the input matrix, leading to an overlap between low-rank approximations and matrix factorization techniques. Overall, it is a popular tool for many real-world problems in both scientific [44, 45] and enterprise domain such as clustering [46, 47], imputation [43, 48], background separation [49, 50], etc.

Here, we provide an overview of the framework for understanding matrix factorization (“low-rank approximation”) and tuning the various parameters on this framework for day-to-day needs of handling different domain observations. For the latter, we use the concept of physical constraints such as sparsity, spatial smoothness, robustness to noise, symmetry, etc. that match the physics of the specific problem. We further provide some examples of physical imaging where these constraints are used to match the physics of imaging process and material properties.

**Fig. 1:**
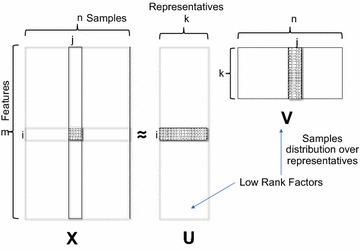
Matrix factorization. The matrix *X* is factored into two smaller matrices *U* and *V*, such that *X* ≈ UV

As a starting point, consider an input matrix $X$ of size *m* × *n*, where ‘*m*’ is the number of features and ‘*n*’ is the number of samples, and a very small number ‘*k*’ called ‘*low*-*rank*.’ Typically, *k* ≪ min(*m*,*n*) may be in the order of 50’s for matrix in size of millions, while *k* less than 10 is typical for matrices of size in a few thousands. It is common in the machine-learning literature to use features, attributes, dimensions, and metrics interchangeably; here, we will consistently use the term ‘features.’ In Fig. 1 there is a pictorial representation of the matrix factorization process with two low-rank factors.

In the case of scientific data, the input matrix can be the hyperspectral data acquired by a wide range of spectroscopic techniques, where signal in each of the *n* spatial points represents a spectrum of length *m*, containing information about local properties. The features in this case correspond to the spatial grid on which measurements are performed (i.e., (*x*,*y*) or (*x*,*y*,*z*)), whereas samples correspond to wavelength, energy, voltage, mass-to-charge ratio, etc. In the case of linear unmixing, the matrix *U* will be interpreted as consisting of *k* endmembers *w*_*i*_(**R**) and *V* as the loading maps *a*_*i*_(**x**).

There are many interpretations for matrix factorization. One consistent view among researchers is the equivalence of matrix factorization to soft clustering [51] with *k* representatives and distribution of every sample over these representatives. Given a matrix *X* of size *m* × *n* with *n* samples of data, where each sample has *m* dimensions, matrix factorization generates *k* representatives as left low-rank factor *U* of size *m* × *k* and the right low-rank factor *V* of size *k* × *n* provides the distribution of every sample among these *k* representatives. That is, consider a sample *j*, if the weight of the 2nd entry is more than 5th entry of the *V* matrix, the sample *j* is associated more with the 2nd cluster over the 5th cluster. This definition is also consistent with the soft clustering of determining ‘*k*’ clusters [51]. Matrix factorization is also a dimensionality reduction technique as it reduces the sample dimension from *m* to *k* in the space of *U*. That is, given the input matrix *X* of size *m* × *n*, we produce a matrix *V* of size *k* × *n* where *k* ≪ *m* and hence the name “*dimensionality reduction*.” For the rest of the paper, we will address matrix factorization mainly as a “*dimensionality reduction*” [52, 53] technique.

One challenging problem in unmixing is determination of the number of endmembers *k*. Ideally, a choice of good *k* is that every point **x** in the loading map *a*_*i*_(**x**) is exactly representable as a combination of the k endmembers *w*_*i*_(**R**). The trivial solution that satisfies this condition is *k* = rank(*X*), where rank is the number of non-zero eigenvalues of the matrix *X*. We are looking for a non-trivial *k* ≪ min(*m*,*n*), that best fits the matrix *X*. Typically, in practice, we increment *k*, until we find the results meaningful. Incrementally updating the number of endmembers and the obtaining loading maps for lower number of endmembers is not computationally expensive. In the scientific domain, we are expecting the number of endmembers typically to be small, i.e., < ~ 10. To statistically evaluate the quality of the unmixing, we may utilize the dispersion coefficient method explained by Kim and Park [54] in the matrix factorization context. There are also other approaches [55] based on information criterion such as Akaike information criterion (AIC) or Bayesian information criterion (BIC) and the elbow method based on law of diminishing advantages [56]. For domain scientists, this problem is akin to one of fitting a model (e.g., a polynomial of order *n*) to data—in those cases, information criterion approaches allow one to apply a penalty on the polynomials of higher order (due to larger available degrees of freedom) that must be overcome for models with higher *n* to be preferred over those with lower *n*.

## Matrix factorization framework (MFF)

The key questions that arise from the previous sections are (a) How does one define the approximation *X* ≈ UV? (b) How to incorporate the properties of the input data *X*, for e.g., positive numbers? (c) How can specific domain knowledge—such as, e.g., the representative spectra should be spatially correlated, it’s a matrix of signals, etc. be incorporated? Most of these questions are addressed in matrix factorization process as one of the following: (refer to Table 1 for details of notations or definitions in this section).

1. *Similarity function X* ≈ UV. Even though UV corresponds to the linear unmixing $\sum\nolimits_{i = 1}^{k} {a_{i} \left( {\mathbf{x}} \right)w_{i} \left( {\mathbf{R}} \right)}$, defining the similarity of UV to *X* is important. For example, it can be an entry-wise closeness of UV to *X* or alternatively the closeness at the individual spectra. That is, every row of UV to individual vector parameter variable **R**.
2. Properties of the input data can be a *hard constraint* on *U* and *V*. For example, the product of two non-negative matrices will always be positive.
3. Characteristics of the data will either be a hard constraint or a *soft constraint* imposed as a regularization. In practice, hard constraints are computationally expensive, and regularization provides good interpretability. Sometimes, for very large matrices enforcing hard constraint might take days to weeks and would require running on distributed supercomputing clusters [24]. The importance of the regularization is always defined through positive regularization constants—the higher the value, the higher the importance. The preference among the conflicting soft constraints is expressed through the values of the corresponding regularization constant. There are scientific libraries such as mlrmbo [57] and hyperopt [58] that help domain scientists determine the values of these regularization constants based on a grid search, line search, random search, or Bayesian optimization techniques.
4. The product of factors can be transformed using a *transformation function f*. For example, a sigmoid function for a Boolean input matrix, or a rounding function in the case of integer input matrix.
5. Preprocessing on the input matrix to generate *X*. For example, a standard practice in microscopy images is to apply a Fast Fourier Transform (FFT). Mean centering is another popular preprocessing step for PCA. Similarly, normalization to generate the matrix *X* in the range of [− 1,1] or [0,1] is another common preprocessing technique.
6. Finally, a less common but an observed practice is providing different weights to the samples. For example, as part of the preprocessing step we assume some engineered features that are augmented to provide better information. Such augmented features will have a different weight towards the observed or measured features.

**Fig. 2:**
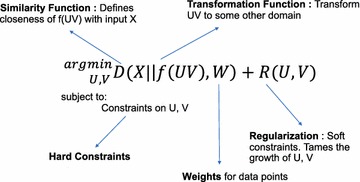
Matrix factorization framework

Figure 2 presents these different control knobs, which are parameters of the matrix factorization process.

The above framework [59] offers a unified way of understanding many dimensionality reduction techniques such as singular value decomposition (SVD), principal component analysis (PCA), non-negative matrix factorization (NMF), and others needed for multivariate analysis of various multidimensional data. Also, it provides the ability to incorporate the physical constraints that govern the underlying process using the above defined parameters. As an example, we will explain the standard PCA and NMF, that is used in the interpretation of microscopy data.

Below in Table 2 we provide some common realizations of the different parameters encountered in Fig. 2.

**Table 2:** Some common realizations of matrix factorization framework parameters

Parameters	Some common realizations
Similarity functions	Frobenius norm, KL-divergence
Transformation function	Logistic function, rounding function
Regularization	Sparsity, spatial
Hard constraints	Non-negativity, orthogonality, sum to one
Weights	Uniform weights
Preprocessing	Mean centering, normalization, log transformation, FFT

### Principal component analysis (PCA)

Principal component analysis (PCA) [60] is a simple, non-parametric method for visualizing high dimensional data. Classical PCA is a linear transform that maps the data into a lower dimensional space by preserving as much data variance as possible. With minimal effort PCA reduces a complex dataset to a lower dimension to reveal the sometimes hidden, simplified structures that often underlie it.

The principal components are the top-*k* eigenvectors of mean subtracted data matrix. That is, consider the matrix A of size *m* × *n*, an input matrix *X* is constructed by subtracting the mean of all the *m* features from each of the *n* samples. We then perform the singular value decomposition (SVD) of the matrix *X*. The eigenvalues of the top-*k* eigenvectors are considered as the principal components of matrix *A*. The above process can be explained in the matrix factorization framework as below.

3 $${\text{subject}}\;{\text{to}}\quad \begin{array}{*{20}l} {\mathop {min}\limits_{{U,\Sigma ,V}} \left\| {(A - \mu ) - U \Sigma V^{T} } \right\|_{F}^{2} } \\ {U^{T} U = I} \\ {V^{T} V = I} \\ {\Sigma \;{\text{is}}\;{\text{a}}\;{\text{diagonal}}\;{\text{matrix}}} \\ \end{array}$$

From the above formulation (3), for PCA we can map the parameters of the MFF, the optimization problem has Frobenius norm as the similarity measure with orthogonality constraints on the factors, where *I* is an identity matrix of size. PCA performs mean subtraction as preprocessing and considers uniform weights for all the data points.

In PCA, the orthogonality of the factors is rigid and can result in having negative values on the factors restricting its interpretability. For example, *V* cannot be interpreted as probability distribution, because of negative values. In such scenarios, we consider using non-negative matrix factorization (NMF).

### Non-negative matrix factorization (NMF)

NMF [61] is the problem of decomposing the input matrix *X* into two non-negative factors *U* and *V* such that *X* ≈ UV. NMF is popular among scientist for spatially resolved spectral analysis, defined as finding *k* ≪ *m* basic spectra (basis functions that change gradually with composition, in terms of structure and intensity), such that all the $m$ measurements can be explained as a mixture of the *k* basic spectra.

Formally NMF can be defined as,

4 $${\text{subject}}\;{\text{to}}\quad \begin{array}{*{20}c} {\mathop {min}\limits_{{U,V}} \left\| {X - UV} \right\|_{F}^{2} } \\ {U \ge 0,V \ge 0} \\ \end{array}$$

In the case of NMF, the common similarity measure is Frobenius norm as in the above formulation (4) and KL-divergence. We are enforcing hard non-negative constraint which means every element in the factors *U* and *V* will be zero or above, and all the samples are uniformly weighted.

#### Sparsity

We often know that the number of endmembers that participate in a particular point on the abundance is sparse, i.e., limited. Consider the distribution for a particular pixel, say 3, on the abundance map from matrix *V* among 4 endmembers could have been [0.48 0.49 0.015 0.015]. The NMF model allocated an insignificant value 0.015 for endmembers 3 and 4 so that it can reduce the overall objective error of the optimization function. But for the domain scientist it can be difficult to delineate these insignificant values. We can overcome this difficulty by enforcing the maximum number of participating endmembers for every pixel in the abundance map. However, it is computationally very expensive to enforce this hard constraint, and instead we use an $\ell 1$—regularizer [25]—a soft constraint for the model to ignore insignificant value on the *V* matrix as follows.

5 $${\text{subject}}\;{\text{to}}\quad \begin{array}{*{20}c} {\mathop {min}\limits_{{U,V}} \left\| {X - UV} \right\|_{F}^{2} + \lambda \left\| V \right\|_{1} } \\ {U \ge 0,V \ge 0} \\ \end{array}$$

#### Spatial smoothing

It is generally observed that the mixture of endmembers around a particular point will be similar. That is, in a 128 × 128 target, the mixture among the neighboring pixels such as (*x *− 1,*y*), (*x* + 1,*y*), etc. around a given (*x*,*y*) is likely to be similar. To enforce this spatial smoothness, we utilize the spatial regularization [62] in MFF. The NMF with spatial regularization can be formally defined as

6 $${\text{subject}}\;{\text{to}}\quad \begin{array}{*{20}l} {\mathop {min}\limits_{{U,V}} \left\| {X - UV} \right\|_{F}^{2} + \lambda _{1} \left\| V \right\|_{1} + \lambda _{2} \left\| {VLV^{T} } \right\|_{F}^{2} } \\ {U \ge 0,V \ge 0} \\ \end{array}$$

In the above formulation (6), *L* is a similarity matrix constructed out of the input matrix among 16,384 pixels. That is, we consider the pair-wise similarity among 16,384 × 1535 matrix that results in a 16,384 × 16,384 symmetric matrix with diagonal elements being zero. By providing this additional information, we are incorporating the neighborhood information implicitly into the matrix factorization process through the regularization constants *λ*_1_ and *λ*_2_.

Further, if all the data are normalized and in a similar range and if *λ*_2_ > *λ*_1_, we are informing the MFF that spatial properties are more important than sparsity. On the one hand, choosing a very low *λ*, may not have any impact on the model at all. On the other hand, a high *λ*, can result in numerical errors and result in infinity, undefined values, or yielding same values across all matrix elements in factors. It is always better in practice to start with relative low regularization values such as 0.001 and increasing in different steps till we obtain a desired value. For example, in this model (6) with spatial smoothness and sparsity, sparsity is relatively an easier constraint over spatial smoothness. Thus, it is preferable to start with a non-zero *λ*_1_, proceed with identifying a good parametric value, and only then tune *λ*_2_. It is important to observe that *λ*’s are always non-negative. Additionally, there are scientific libraries such as mlrmbo [57] and hyperopt [58] that can aid this determination, with automated approaches to determine the values of these regularization constants.

MFF can incorporate different physical constraints during matrix factorization such as sparsity, spatial smoothness, non-negativity, etc. In this paper, we are using the open source implementation from https://github.com/ramkikannan/nmflibrary. Kannan et al. [50] provide the details about the implementation in their paper. We would like to conclude modeling different popular matrix factorization techniques under MFF in Table 3.

**Table 3:** Modeling of different dimensionality reduction techniques on MFF

Matrix factorization	Transformation	Constraints	Regularization	Weights	Similarity
SVD [63]	None	Orthogonal *U*^*T*^*U* = *I* *V*^*T*^*V* = *I*	None	Uniform	Frobenius
PCA [64]	None	Orthogonal *U*^*T*^*U* = *I* *V*^*T*^*V* = *I*	None	Uniform	Frobenius
NMF [65]	None	Non-negativity $U \ge 0, V \ge 0$	None	Uniform	Frobenius
pLSI	None	Sum to 1	None	Uniform	KL-divergence
Sparse NMF [25, 66]	None	Non-negativity $U \ge 0, V \ge 0$	ℓ1 on *V* ||*V*||_1_	Uniform	Frobenius
Bounded [26, 27]	None	Bounded entries in the low-rank approximation $\alpha < UV < \beta$	None	Uniform	Frobenius

## Domain-specific applications

**Fig. 3:**
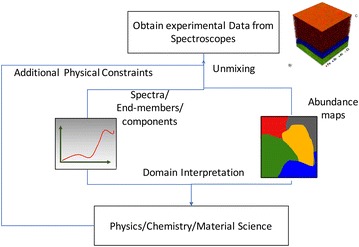
Unmixing workflow for domain scientists

In this section, we begin with the illustrative workflow in Fig. 3 of the unmixing process followed by scientists.

The process begins when a scientist generates some multidimensional imaging data, typically (but not always) in a spatially resolved fashion. Each point or pixel consists of a spectra, and the aim is to unmix this multidimensional dataset into a smaller number of constituent spectra, to aid in interpretation and to speed up visualization with minimal information loss. After preprocessing of the data (which can be either simple or elaborate), the unmixing algorithm is applied, and produces endmembers and abundance maps which are then interpreted by the domain expert. When the abundance maps and the components lack physical meaning, scientists may retry the unmixing by imposing physical constraints as necessary. For e.g., if the spectra from PCA have negative values, they will introduce non-negative constraints through NMF. This process is iterated till the obtained endmembers and the spatial maps are physically justifiable.

Listed in Table 4 below are some examples of the scientific applications and the potential constraints of matrix factorization approaches. The approach lends itself directly towards applications where measured spectra necessarily arise from mixing of multiple components in an additive fashion. Given variations in the strengths of these mixings, e.g., across spatial or temporal domains, the captured spectra will constitute the matrix to be factored using MFF approaches. The goal in these tasks is usually to determine the constituent (‘purest’) spectra, corresponding to, e.g., ideal crystal phases (X-ray crystallography), particular chemical species (chemical imaging such as time-of-flight secondary ion mass spectrometry, ToF-SIMS), specific electronic structures (scanning tunneling microscopy and current imaging tunneling spectroscopy, STM and CITS), etc.

**Table 4:** Some scientific applications and potential constraints to matrix factorization approaches

Scientific applications	Data dimension	Input vector	Constraints
ToF-SIMS	3D	2D (spatial × mass spectrum)	Non-negativity
STEM (phase analysis by sliding FFT)	4D	2D (spatial × FFT spectrum)	Non-negativity
STM	3D	2D (spatial × tunneling spectrum)	Non-negativity, sum to 1
X-ray microscopy	3D or 4D	2D (spatial × *Q* spectrum)	Non-negativity, sum to 1, orthogonality
Raman spectra (AFM)	None	2D (spatial × Raman spectrum)	Non-negativity

Note that sparseness and spatial smoothness constraints discussed in the text are generally applicable to each of the listed methods

Specific constraints are applied based on known physical facts, for instance, chemical mass spectra in ToF-SIMS are always positive (negative concentration of a species is not defined). Similarly, analysis of electron energy loss spectra (EELS) also implies positivity on all factors and abundances. The sum-to-one constraint on the abundances also arises from basic scientific considerations. Assuming that the measured spectra are linear superpositions of constituent spectra, then each abundance is effectively a percentage spectral weight, with the coefficients summing to one. This is true for chemical spectra, X-ray diffraction, etc.

Note that for the *qualitative* analysis of features commonly seen in CITS curves (such as presence/absence of kinks, interpeak separation, and ratio of peak heights) the sum-to-one requirement may be omitted, as long as a non-negativity constraint is imposed. An additional complication arises in determining the optimum number of components. In many cases this value is unknown *apriori*, but can be easily estimated based on similarity of resulting components when the unmixing is computed for increasingly more components: beyond some threshold *k* components, additional components will begin to appear similar to other components.

In addition, sparsity and smoothness constraints can be used for analysis of spatial distribution of defects and, in some specific cases, shapes of spectral curves. The main idea behind applying sparsity constraints to abundance maps is a relatively low probability of several phases being observed simultaneously in one pixel. For example, it is very unlikely that more than one type of structure or chemical phase can be present within a pixel whose size is around several angstroms. By the same token, there are certain scenarios, for example in the chemical and STM spectroscopies, in which the chemical or electronic state associated with one endmember (e.g., defect-induced localized state) may not appear at the same value of energy in other endmembers (e.g., in a gapped superconducting phase). The smoothness constraints, meanwhile, imply that the mixture of endmembers around a particular pixel in the abundance maps do not vary strongly.

For a microscopic experiment, smoothness is generally expected to be obeyed when the achievable lateral resolution in the imaging data is larger than the pixel size in the same dataset. That is, it is generally not possible that individual pixels can be surrounded by pixels of a different factor, given finite probe size and associated convolution of the signal across multiple pixels. At the same time, the imposition of the sparsity constraint requires domain knowledge. In some cases, multiple mechanisms (spectra) can co-exist, but in many cases, they cannot. As one example, unmixing distinct electronic phases from I–V data with sparsity constraint implies that at any one pixel, there cannot be contribution from multiple competing transport phenomena (such as Ohmic and Schottky emission). Moreover, from a fundamental physics perspective smoothness is enforced because interfaces separating distinct phases tend to be smooth to lower energy, and sparsity comes from the fact that, e.g., multiple structural phases cannot co-exist in the same location.

In the section below, we deal with the various scientific applications of the MF approach.

### Time-of-flight secondary ion mass spectrometry (ToF-SIMS) data

Time-of-flight secondary ion mass spectrometry (ToF-SIMS) is a chemical imaging technique, widely used for chemical characterization of organic and inorganic systems. In ToF-SIMS, focused ion beams are used to release material species from the studied sample. Those ions are further accelerated in electric field and analyzed using mass detector [15, 67]. Using multiple ion guns, ToF-SIMS allows investigations in the bulk of the sample; in this case the results represent a 4-dimensional data cube with three spatial (*X*, *Y*, and *Z*) and one spectral (mass-to-charge) dimension. Non-negative matrix factorization (NMF) can be used as a basis for automated interpretation of this data. In this case, each mass spectrum is considered as a mathematical vector *X*_*i*_, in spatial point *I*, which is deconvoluted as linear combination of limited number of non-negative endmembers *w*_*j*_ and noise term *N*_*i*_.

7 $$\begin{array}{*{20}l} {X_{i} = \sum\limits_{i} {A_{{ij}} w_{j} + N_{i} } } \\ {{w}_j \,> 0, {A}_{ij} > 0,} \\ \end{array}$$

where *A*_*ij*_—abundance coefficients.

Non-negative matrix factorization can be used for automated analysis and interpretation of the hyperspectral data acquired by wide range of spectroscopic techniques, where signal in each point represents a spectrum, containing information about local properties. In this case, multidimensionality and size of the resulted data render more traditional methods of data analysis substantially difficult.

### ToF-SIMS 2D imaging

**Fig. 4:**
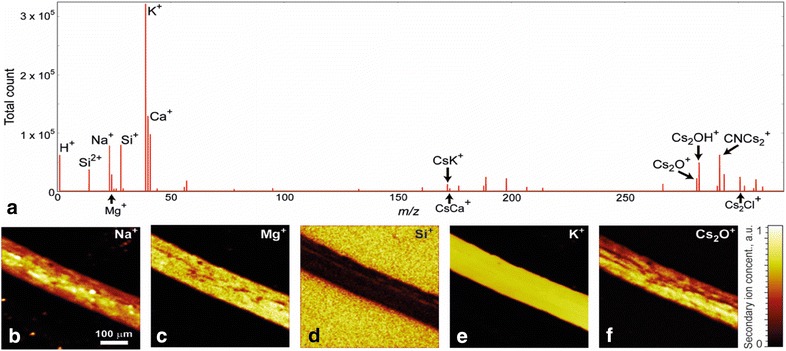
**a** Averaged mass spectrum of Arabidopsis root. **b**–**f** Maps of the spatial distribution of elements

In this section, we compare the output of application of NMF and PCA algorithms on ToF-SIMS experimental data. The details about the experiment and the procedure of the ToF-SIMS data preparation for factorization can be found in ref [68]. Briefly, ToF-SIMS chemical imaging was performed on an Arabidopsis root sample placed on an SiO_2_ substrate. After necessary relevant preprocessing, we obtained a mass spectrum of length 1535 over 128 × 128 pixel target. We constructed this a matrix of size 1535 × 16,384 as a spectrum of every pixel of the target image. The maps of the spatial distribution of various elements, along with the averaged mass spectrum, are shown in Fig. 4.

**Fig. 5:**
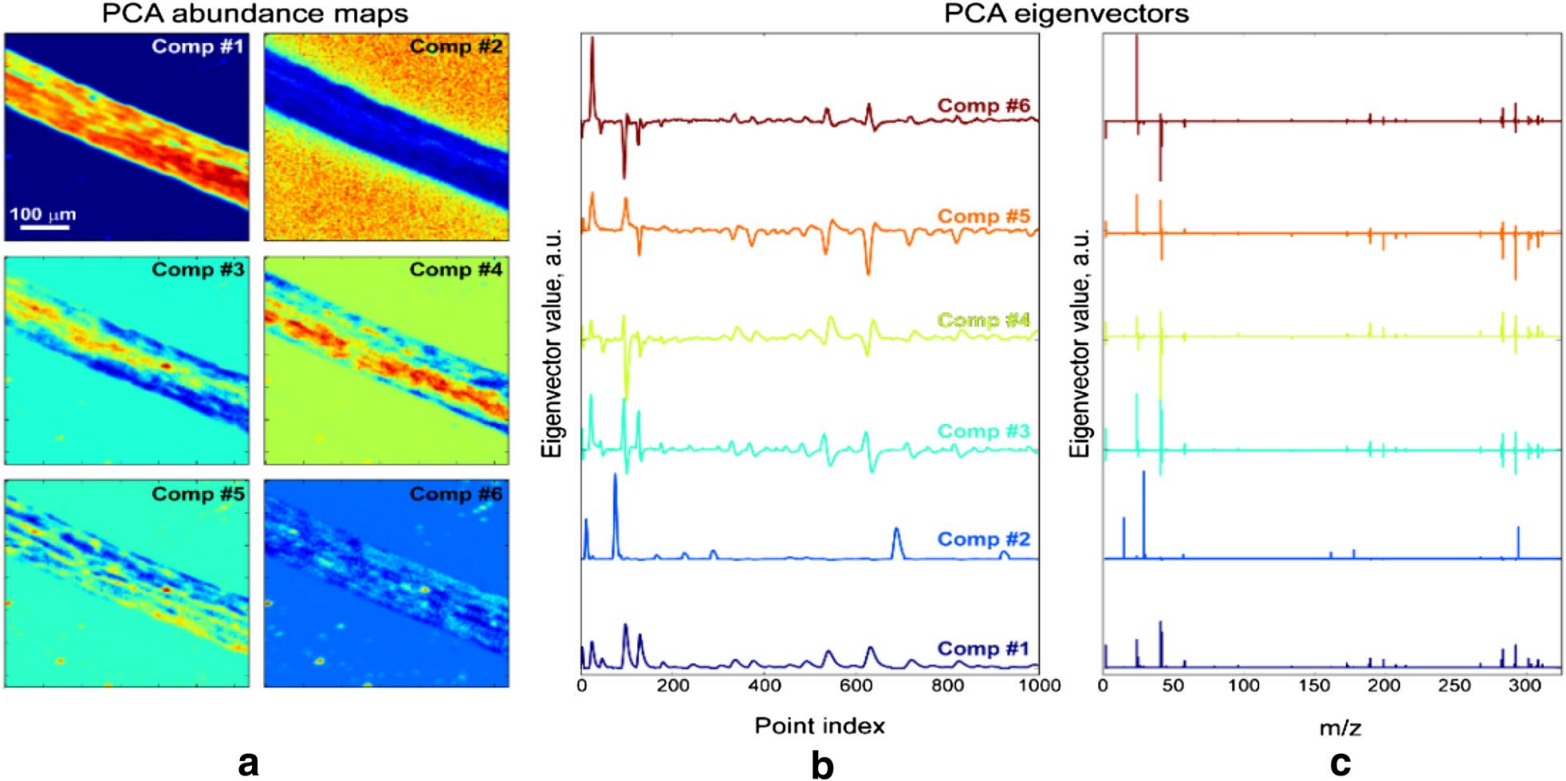
Principal component analysis performed on ToF-SIMS data. **a** Abundance maps and **b**, **c** eigenvectors plot vs **b** point index and **c** mass-to-charge ratio

We first performed PCA analysis of this data, with the results shown in Fig. 5. This analysis shows there exists significant deviations in the chemistry within the root. To understand these results, we note that the mass spectrum in each point represents a linear combination of eigenvectors (Fig. 5b, c) with loading coefficients coded by color on the loading abundance (Fig. 5a). For example, component #1 shows averaged mass spectrum of the root, without the characteristic Si peaks. On the other hand, component #2 shows only peaks characteristic for Si (Si^+^, Si^2+^, Si_2_^+^, etc.), which can be found outside the root (see (Fig. 5a, map #2)). Component #6 most likely is responsible for some kind of contamination, which is sparsely distributed over the root and substrate and contains higher concentrations of Na. However, analysis of other components is hampered by the view of their eigenvectors, which show both positive and negative values. This is one the fundamental shortcomings of the PCA, where eigenvectors are built to be orthogonal. However, this is physically meaningless, since the count signal in mass spectrum is non-negative.

**Fig. 6:**
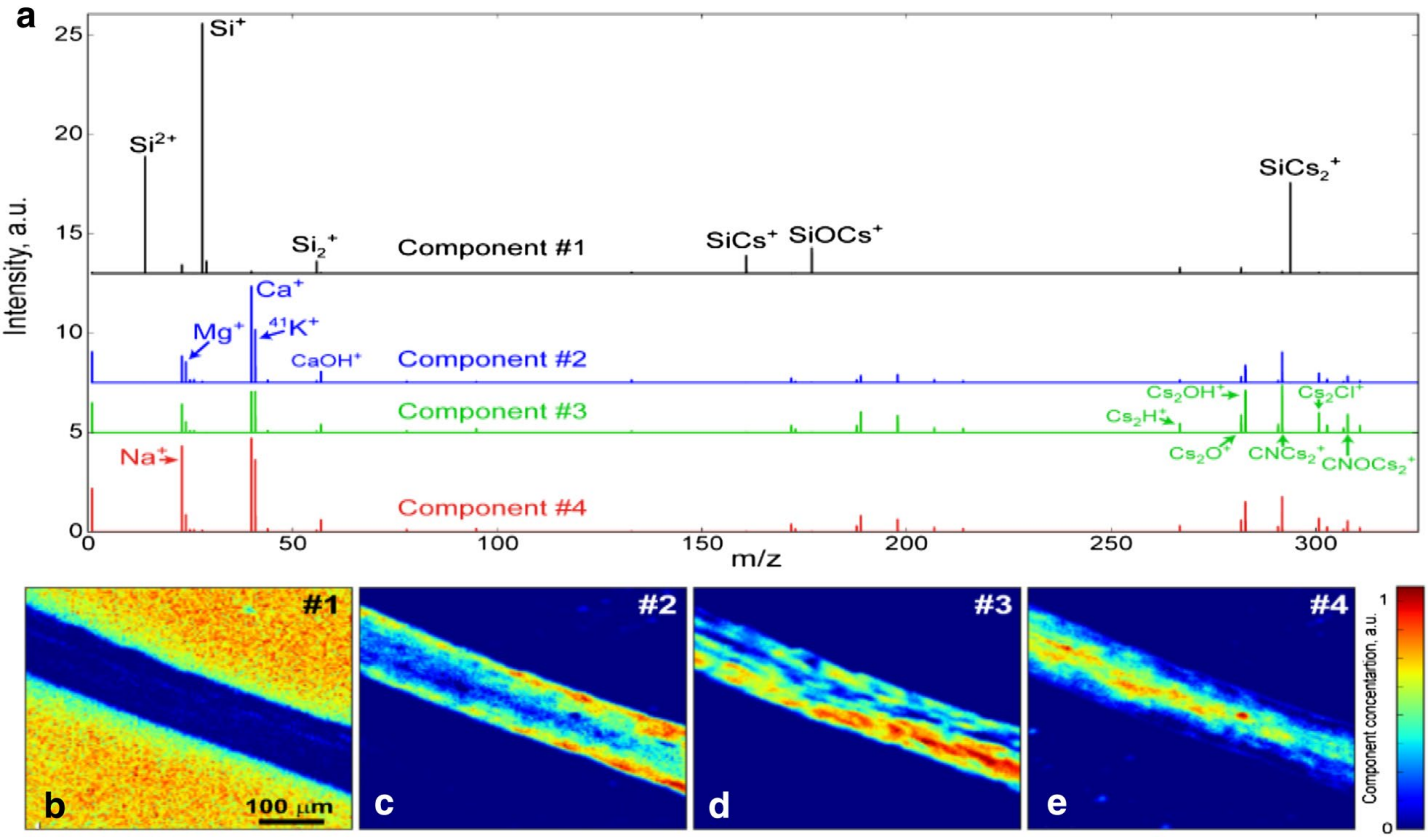
Results of non-negative matrix factorization (4 components). **a** Detected spectral endmembers and **b**–**e** corresponding abundance maps

The results of the NMF over ToF-SIMS data are presented in Fig. 6. The best output was found for the unmixing on 4 components. Unlike PCA, endmembers in NMF are presented in the form of classical mass spectra (Fig. 6a) with abundance maps (Fig. 6b–e) showing their concentration at each point. To check accuracy of the data unmixing we compare real data with data restored from four NMF components. Component #1 clearly shows mass spectrum of the SiO_2_ substrate, and all peaks can be easily identified. This agrees with its spatial distribution outside the root (Fig. 4d). On the contrary, other components were mostly localized inside the root, and show variations in its chemistry. Component #2 shows regions with significant amounts of the base inorganic elements (Mg^+^, Ca^+^, K^+^, etc.). Much higher intensities of small molecules (mass range 150 ÷ 350 *u*) as well as Cs_2_O^+^, Cs_2_OH^+^, CNCs_2_^+^ were found in the component #3, which is most likely related to regions of concentration of organic compounds and growth hormones. Finally, component #4 demonstrates regions with the higher Na concentrations within the root, which is in a good agreement with its map of spatial distribution (Fig. 4e).

After exploring the differences between NMF and PCA, we further explore the possibility of incorporating two common physical constraints—(a) sparsity and (b) spatial smoothing in the MFF, for this dataset.

**Fig. 7:**
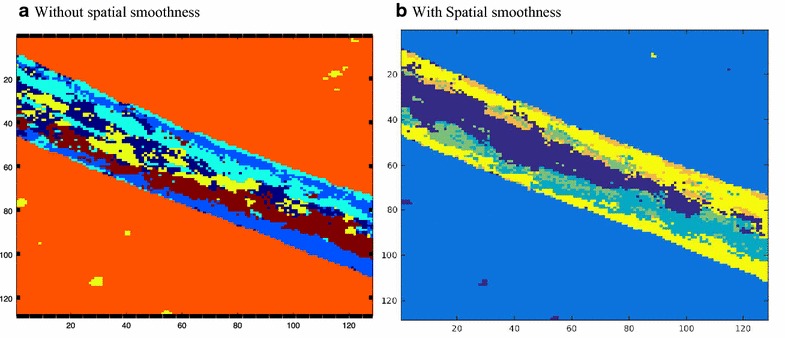
NMF results showing abundance maps **a** without and **b** with spatial smoothness constraints added

In Fig. 7, we present the NMF result with and without spatial smoothness for the ToF-SIMS data of a particular component. We can observe from Fig. 7b that the number of different non-zeros around a particular pixel is smaller than that of Fig. 7a. That is, in Fig. 7b, the probability of having the same neighboring pixels around a given pixel (*x*,*y*) is higher.

In the following sections, we will study enforcing non-negativity constraints in detail for different types of spectroscopic experiments.

### ToF-SIMS 3D

Linearity and non-negativity of endmembers in the case of ToF-SIMS, as well as any mass spectrometry technique has perfect physical sense, as measured mass spectra represent a linear combination of responses of various chemical species belonging to the studied sample.

Here we demonstrate NMF for investigations of the chemical composition of an 80-nm-thick BiFeO_3_ (BFO) ferroelectric thin film, grown on 10 nm LaSr_0.5_Co_0.5_O_3_ (LSCO) buffer layer on a LaAlO_3_ (LAO) substrate. ToF-SIMS investigations of the film were performed using TOF. SIMS 5 (ION-TOF, Germany) instrument with Bi-ion primary gun and Cs-sputtering gun. Measurements were performed in positive ion detection mode, which allowed the detection of metal ions, in addition to that cluster formed with cesium, were used for the identification of some negative species (e.g., Cs_2_O^+^ for O^−^, Cs_2_OH^+^ for OH^−^, and Cs_2_Cl^−^ for Cl^−^).

Investigations have been performed in the bulk of the sample, which allowed to study local distribution of the chemical composition through the thickness of the BFO film, LSCO layer, and part of the substrate. Details about the film properties and corresponding ToF-SIMS investigations can be found in refs [69, 70].

**Fig. 8:**
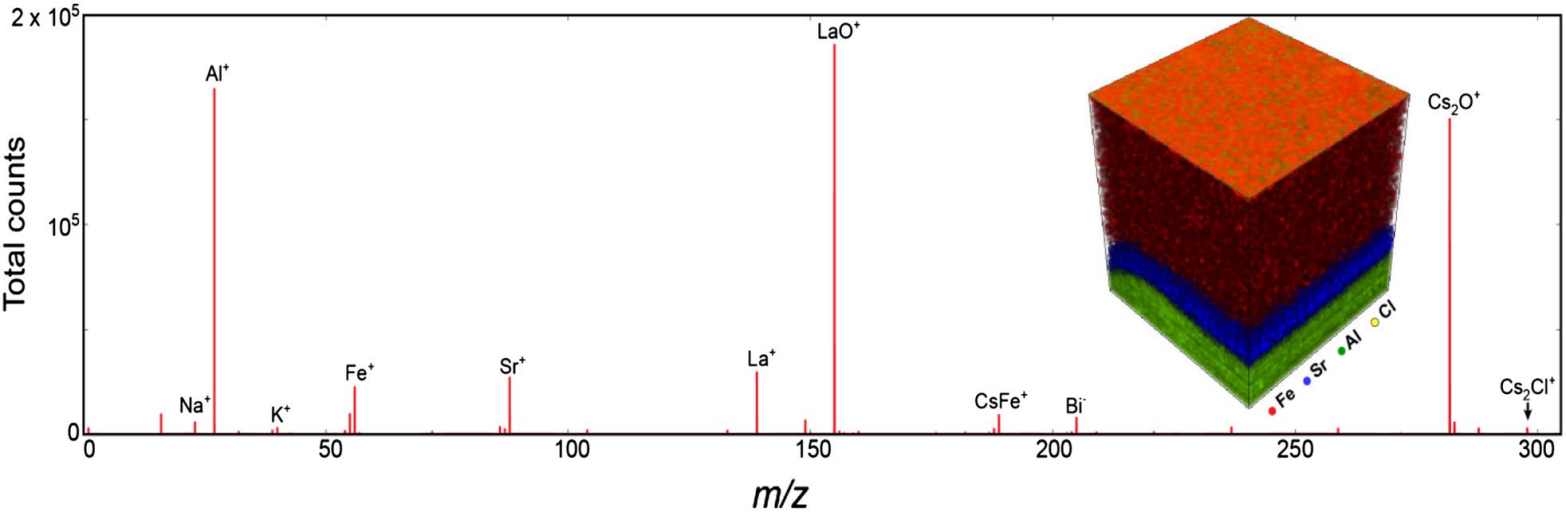
ToF-SIMS investigations of BFO thin film on LSCO buffer layer and LAO substrate. Averaged mass spectrum and 3D spatial distribution of Fe^+^, Sr^+^, Al^+^, and Cs_2_Cl^+^ ions (inset) [70]

Figure 8 shows the mass spectrum averaged over whole dataset and also shows presence of all base elements of BFO, LSCO, and LAO (Al^+^, Fe^+^, Sr^+^, La^+^, Bi^+^), as well as species from adsorption layer (Na^+^, K^+^, and Cs_2_Cl^+^). We performed NMF for interpretation of the 3D spatial distribution of all detected chemical species. Procedure of the ToF-SIMS data preparation for factorization can be found in ref [68].

**Fig. 9:**
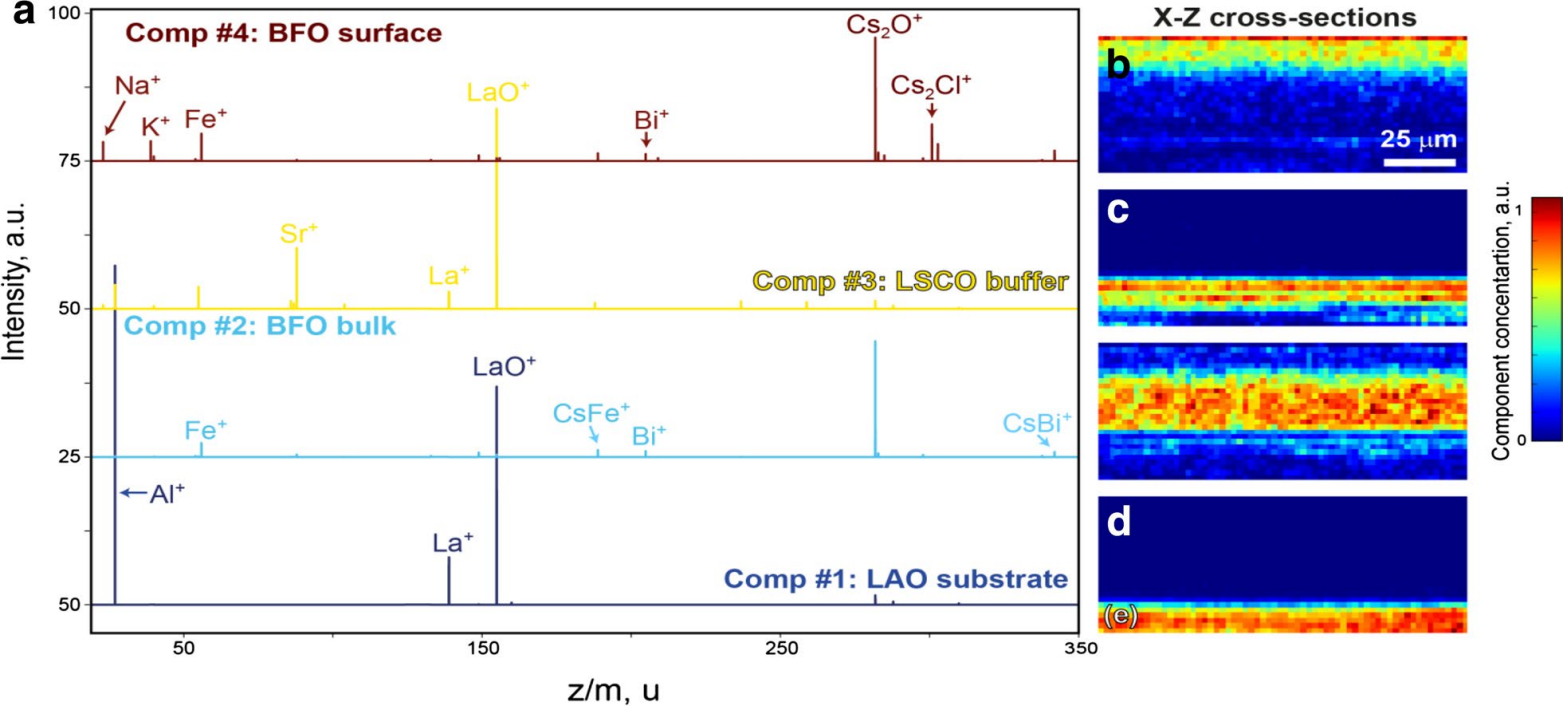
Results of NMF performed on 4-dimensional ToF-SIMS data. **a** Calculated endmembers, **b**–**e** X–Z cross sections of corresponding abundance maps

Our analysis showed superior results for factorization with 4 endmembers, with the corresponding endmembers and cross section of 3D abundance maps plotted in Fig. 9. These data can be used for results interpretation. Specifically, the mass spectrum of component #1 demonstrates pronounced peaks of Al^+^, La^+^, and LaO^+^ and localized at the bottom of the scan (Fig. 9e), thus is responsible for LAO substrate. Component #3 represents LSCO buffer layer—it shows peaks of La^+^, Sr^+^, and LaO^+^ and exists in narrow stripe in between BFO and LAO (Fig. 9c). Bi^+^ and Fe^+^ thin film can be found in both components #2 and #4, however their mass spectra are significantly different.

Component #2 is responsible for bulk BFO signal (Fig. 9d) and shows weaker signals of pure Fe^+^ and Bi^+^, than component #4 related with BFO surface. This is related with measurement technique, where Cs is used for the sputtering and it forms clusters with many of the released species. Consequently, in bulk scans some Fe^+^ and Bi^+^ ions form CsFe^+^ and CsBi^+^ clusters and decrease signal of the pure ions in the mass spectra. In addition, component #4 demonstrates the presence of elements from the adsorption layer (Na^+^, K^+^, Cs_2_Cl^+^), which are localized on the sample surface (Fig. 9b); this is in a good agreement with previous studies [68].

To summarize, enforcing non-negativity constraint in the MFF, provides powerful capabilities for automated analysis of the mass spectrometry data acquired from multicomponent system. In this case data analysis is simplified to the interpretation of the limited number of endmembers with known mass spectra and maps of the spatial distribution.

### Scanning transmission electron microscopy (STEM)

The modern-day scanning transmission electron microscopy (STEM) allows atomically resolved imaging of multiple structural and/or chemical phases within a single image, as well as observing transitions between different phases in a series of images [71, 72]. Such experimental capabilities demand development of analytical method for rapid extraction and identification of different phases, and mapping their spatial distribution. Here we describe how the NMF technique can be combined with sliding window fast Fourier transform (FFT) to allow accurate identification and mapping of different structural and chemical phases.

An application of sliding FFT to atomically resolved microscopic images has been discussed in our earlier publications [73, 74]. Briefly, a stack of 2D FFT maps is generated by shifting a window of a selected size across an experimental STEM image such that the entire image is scanned. At each step an FFT map is computed from a region bounded by the sliding window. If we assume that the image structure factor is a linear superposition of the individual constitutive elements, then an application of NMF to the sliding FFT data allows identifying local structure factors (endmembers) and loading maps [73].

**Fig. 10:**
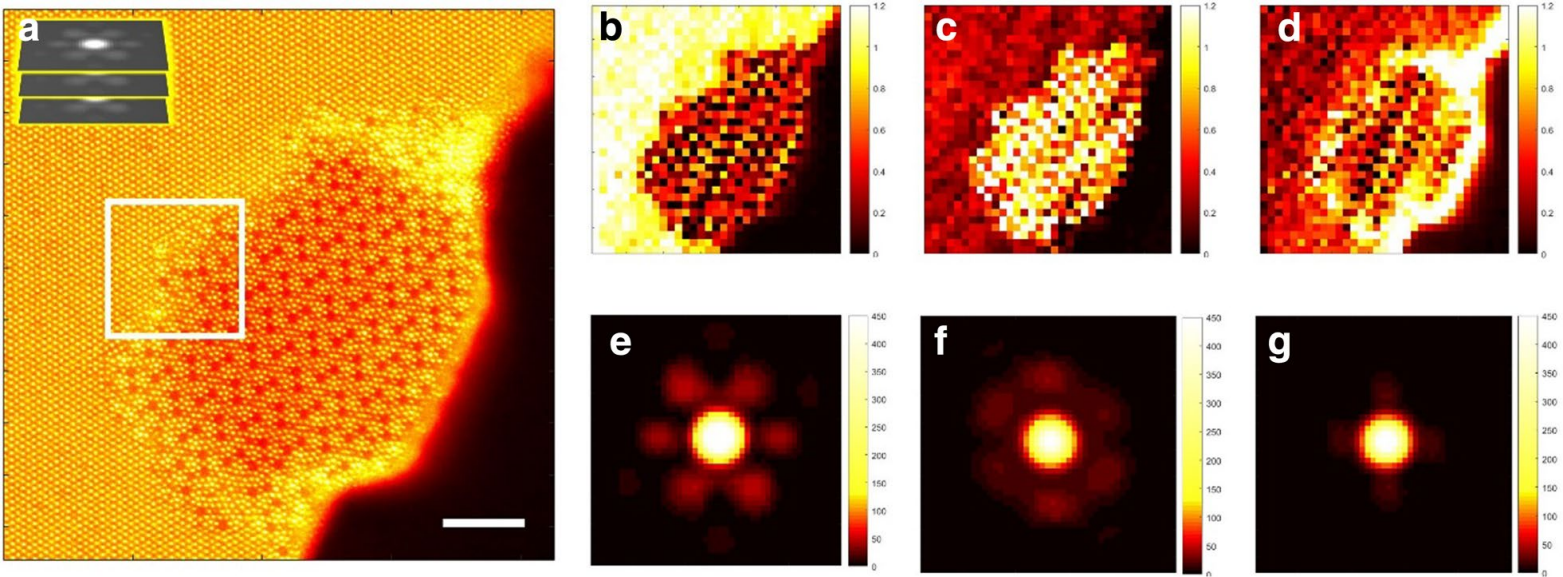
**a** Experimental STEM image of a Mo−V−Te−Nb oxide catalyst. The image size is 2048 px × 2048 px, the width of the window in a sliding FFT is set to 500 px (shown schematically in the figure), and the window step size is 100 px. The top left corner inset shows schematically a stack of 2D FFT images formed during the sliding FFT procedure. The scale bar is 5 nm. **b**–**g** Results of NMF-based decomposition of sliding FFT data over the area in **a** into 3 components. Loading maps (**b**–**d**) associated with endmembers (**e**–**g**). The original image used in **a** is reproduced (adapted) with permission from He et al. [67]. Copyright (2015) American Chemical Society

As a model published elsewhere we consider an atomically resolved image of an oxide catalyst, shown in Fig. 10a [75]. The results of the NMF analysis for the sliding FFT data obtained from this image are shown in Fig. 10b–g. The two chemical phases are clearly identified in the first and second components (Fig. 10b, e and c, f), whereas the third component can be interpreted as due to a presence of interface regions. Therefore, the use of NMF allows to match the physics of diffraction (in the absence of dynamical effects), i.e., that spectra can be deconvoluted linearly, and the fractions must sum to 1. Moreover it shows that image segmentation is possible, although in future this should be done with symmetry-based constraints on the unmixing process (to determine the space group for each phase). This ability to accurately map different chemical phases within a single STEM frame (image) could become especially valuable during analysis of phase transitions observed via STEM in a frame-by-frame manner (STEM ‘movies’). We also foresee that in future a combination of sliding FFT and NMF tools can be applied to scanning tunneling microscopy of quasiparticle interference patterns in strongly correlated electronic materials in which different coexisting phases (and/or different scattering centers) may produce several interference patterns with distinct symmetries within an experimental field of view.

### Current tunneling imaging spectroscopy (CITS)

We next illustrate an application of NMF methods to extracting physics from current imaging tunneling spectroscopy (CITS) of a strongly correlated electronic system. CITS is a mode of operation of a scanning tunneling microscope that allows extracting 3-dimensional (3D) maps of differential tunneling conductance *G *= d*I*/d*U* with sub-nanometer resolution. The value of *G*(*x*, *y*, *U*) in each recorded point (pixel) reflects an electronic density of states on the surface at energy *E* = e*U* [76]. We specifically focus our attention on CITS dataset obtained from a surface of BaFe_2_As_2_ compound with hole doping by Mo substitution (*x* ≈ 0.026) on the Fe sites. This compound could play an important role in discussing mechanisms behind unconventional superconductivity in FeAs-based systems since a superconducting behavior in these materials is observed only at electron doping of the Fe sites by 3d and 4d transition metal atoms but not at hole doping [77, 78].

**Fig. 11:**
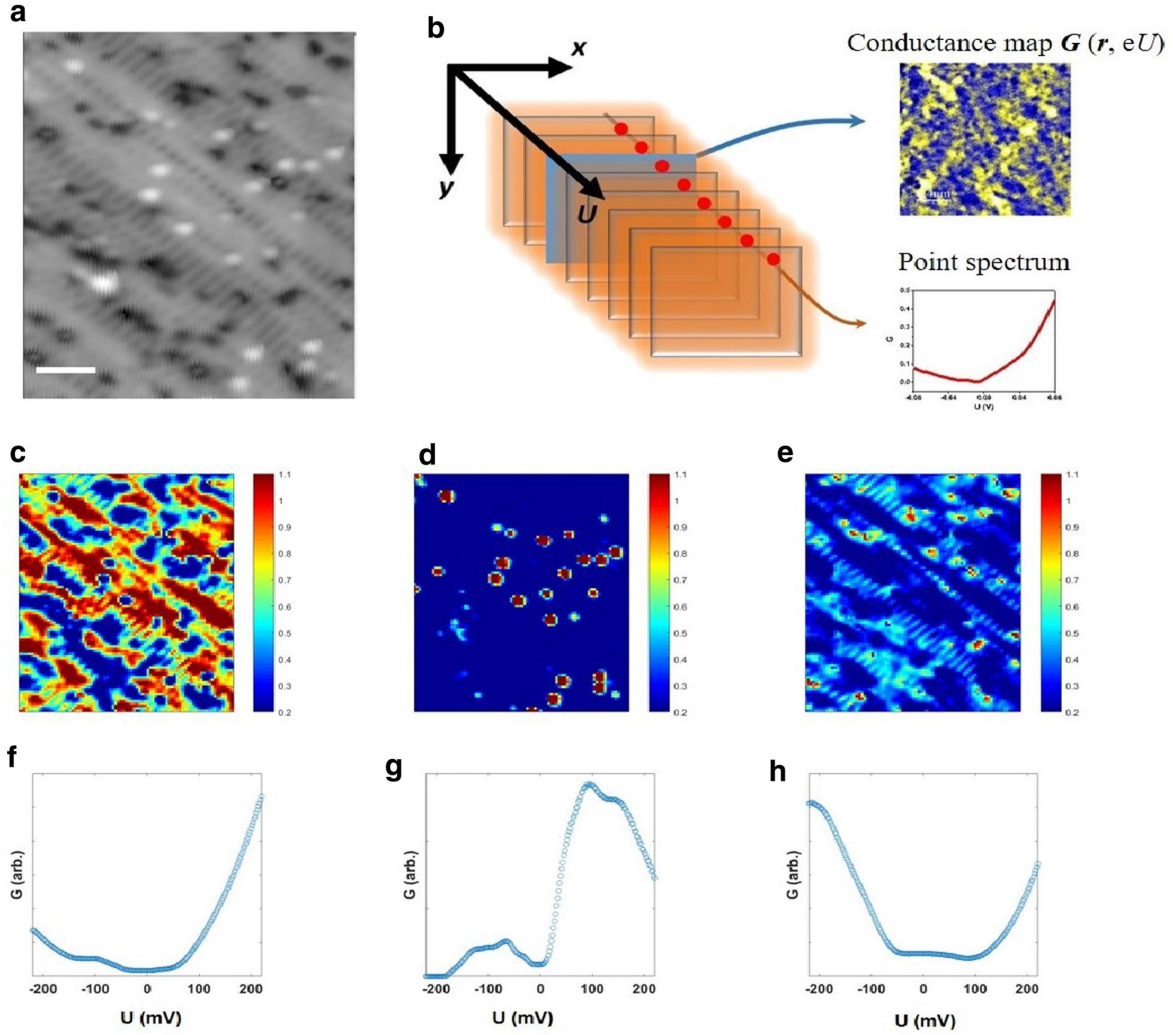
**a** STM topography of Mo-doped BaFe2As2 surface obtained at *T* = 4 K. The scale bar is 5 nm. **b** Schematics of CITS experiment in which a 3D stack of conductance maps G (r, eU) is acquired over STM field of view. **c**–**h** Results of NMF-based decomposition o of CITS data over the area in **a** into 3 components. Loading maps (**c**–**e**) corresponding to spectral endmembers (**f**–**h**). See text for more details

Figure 11a shows a representative STM topographic image of in situ cleaved Mo-doped BaFe_2_As_2_ surface obtained at T = 4 K. The topographic data immediately reveal several characteristic surface features such as a presence of regions with and without a stripe-like surface reconstruction, as well as point-like (lateral size ~ 1 nm) bright blobs and depressions dispersed across the entire field of view. Similar to an earlier analysis of STEM data, our assumption here is that CITS signal can be represented as a linear superposition of currents flowing through each of the available “channels” during the experiment. We next apply NMF to the CITS dataset of the dimensions *x* × *y* × *U* = 80 × 100 × 220 recorded over an area shown in Fig. 11a. The results of the NMF-based decomposition (endmembers and loading maps) into 3 components are Fig. 11c–h. We note in passing that the NMF decomposition into a larger number of components adds only components associated with a noise. Analysis of the loading map in Fig. 11c suggests that the first component is primarily connected to regions without surface reconstruction. The corresponding spectral curve (endmember 1) in Fig. 11f has a characteristic bump at about ≈ − 100 meV and a vanishing density of states at around the Fermi level likely associated with a formation of spin density wave gap below T = 119 K [77]. The second component clearly originates from a presence of point-like protrusions on the surface (Fig. 11d, g). These point impurities produce a well-defined peak in the density of states at ≈ + 100 meV seen in the endmember 2 (Fig. 11g). Noteworthy, such a well-defined feature present in the experimental electronic density of states and an information obtained about its distribution on the surface allows to significantly narrow down a range of defect structures to be considered in either theoretical modeling of the sample’s surface or in spatially averaged spectroscopic experiments. Finally, the third component can be linked to certain depressions on sample’s surface (albeit not all of them) (Fig. 11e, h). There are no pronounced localized states associated with these depressions in the energy range of interest, although they do modify the character of electronic structure around the Fermi level as seen in endmember 3 (Fig. 11h). Overall, such an unprecedented insight into the details of spatial localization of various electronic features acquired through application of NMF method can be crucial for better understanding mechanisms behind emergence/suppression of superconductivity in FeAs system in future studies. It further shows the utility of the method in segmentation into distinct electronic phases (for example, for determining metal–insulator transitions [79]), which is only possible because positivity is enforced.

### Structural X-ray imaging

The accurate determination of structural phases and evolution of epitaxial strain in crystalline thin film heterostructures is one of the most active research areas in structural imaging. The most commonly employed structural probe, namely X-ray diffraction (XRD), provides crucial information on the crystalline state of thin films, ranging from atomic unit cell configuration in each thin-film layer to the crystalline quality or mosaic spread of a thin film. The structural information from XRD is, however, spatially averaged over macroscopic distances of the sample [80]. As such, the structural state as determined by XRD is more suitably described as an ensemble average. Various extensions of XRD into a spatially resolved probe has been pursued in the past, ranging from single crystal X-ray diffraction topography [81] to micro-diffraction [82], the ultimate goal being the determination of the individual structural microstates present in a system. With the advent of third generation synchrotron sources and considerable advances in optics that operate in the hard X-ray regime [83] (from angstrom to subangstrom wavelengths), numerous X-ray diffraction imaging techniques have sprung out [84–86], whose spatially resolving capabilities are most suitable to probing the crystal structure of epitaxial thin films. Despite the photon flux limitations of these techniques, a general consequence of the weak hard X-ray scattering cross sections from matter, the exquisite sensitivity of X-ray diffraction imaging to the atomic structure, all but guarantees datasets with unprecedented complexity and richness in information. Extracting the salient structural microstates of materials from these datasets, invariably requires advanced data mining techniques such as matrix factorization.

Here, we demonstrate the potential of matrix factorization, in particular non-negative matrix factorization, in determining epitaxial strain inheritance in an oxide hetero-structure from full-field hard X-ray diffraction microscopy (XDM).

**Fig. 12:**
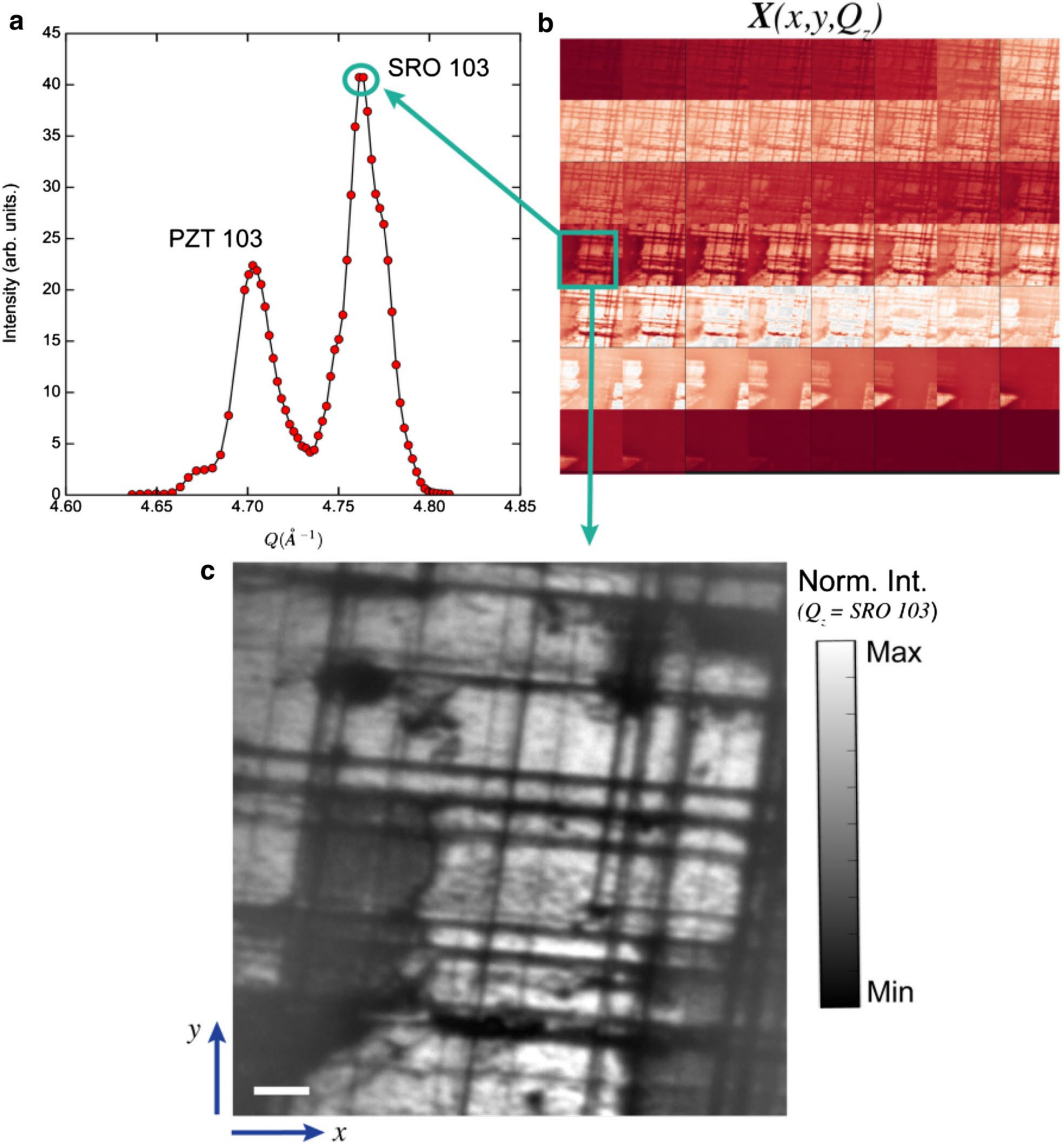
X-ray diffraction scattering and X-ray diffraction Microscopy of (80 nm) Pb(Zr_0.2_Ti_0.8_)O_3_/(50 nm) SrRuO_3_/SrTiO_3_ (001). **a** XRD scan along the (10) truncation rod of SrTiO_3_ (001), showing the PZT and SRO 103 Bragg peaks, Qz is the momentum transfer along the surface normal *z*, at an X-ray energy of 10 keV. **b** XDM images acquired at each Qz point in **a**. The total set of images is denoted by *X*(*x*,*y*,*Q*_*z*_), where (*x*,*y*) corresponding to lateral sample positions and an effective pixel size of 15 nm. **c** A close-up view of an XDM image taken at the SRO 103 Bragg reflection, showing the presence of a network of misfit dislocations (dark lines) that relieve the strain imparted on SRO by the substrate, as well as other regions of the film that appear in dark contrast, indicating the presence of substantial in-plane lattice variations across the SRO layers. Scale bar is 1 µm and the color bar is normalized X-ray diffraction intensity

XDM is a dark field imaging technique which employs a combination of hard X-ray optics to form a real space image of the sample with diffraction contrast. By operating in a Bragg reflection geometry, XDM is sensitive to the full three-dimensional atomic structure of a material with a lateral spatial resolution of ~ 70 nm [87], with structural imaging contrast that is diffraction limited (sub-Å) [86]. One of the simplest operation modes of XDM is by scanning one of the crystal truncation rods of the substrate, to spatially resolve the spatial distribution of the induced epitaxial strain on the different crystalline layers in a hetero-structure (Fig. 12). The XDM dataset originating from the rod scan consists of real space images (Fig. 12b) taken at different *Q*_*z*_ positions along the truncation rod (Fig. 12a), where *Q*_*z*_ is the momentum transfer along the surface normal *z* (see Fig. 12 caption). The resultant XDM dataset, ***X***(*x*,*y*,*Q*_*z*_), therefore depends on image pixel position (*x*,*y*) and *Q*_*z*_, with the image pixels (*x*,*y*) corresponding to lateral sample positions with an effective pixel size of 15 nm (Fig. 12c). As such, ***X***(*x*,*y*,*Q*_*z*_) can be simply interpreted as a spatially resolved XRD, with an XRD intensity *I*(*Q*_*z*_) associated with each sample position (*x*,*y*).

The studied oxide hetero-structure is composed of (80 nm) Pb(Zr_0.2_Ti_0.8_)O_3_/(50 nm) SrRuO_3_/SrTiO_3_ (001), with Bragg diffraction peaks (103 reflection) indicated in Fig. 12a. Due to the large thickness of the SrRuO_3_ (SRO) layers and its in-plane lattice mismatch with the single crystal SrTiO_3_ (STO) (SRO: *a*_pc_~ 3.93 Å, STO: *a*_pc_= 3.905 Å), considerable strain relaxation is expected through the formation of threading dislocations and inhomogeneous spatial distributions in the in-plane lattice constant of SRO [88], resulting in a broadening of its Bragg peak. The presence of these threading dislocation networks in the SRO film is clearly visible in XDM (image taken at *Q*_*z*_ = SRO 103), appearing as dark lines since the presence of rotations in the crystal lattice planes near the dislocations moves the Bragg condition away from its nominal position for the dislocation-free regions of the thin film.

The different structural signatures of strain-relieving mechanisms and spatial distributions of structural phases present in the SRO and PZT layers are encoded in ***X***(*x*,*y*,*Q*_*z*_), and can be extracted by non-negative matrix factorization (NMF). In light of the discussion above, the constraints of orthogonality (SVD, PCA) and linear convexity (pLSI) are not justifiable for an XDM rod scan, since the signal from different structural configurations does not satisfy these constraints, but it does satisfy the constraint of non-negativity, motivating our application of NMF.

Prior to application of NMF, the XDM dataset ***X***(*x*,*y*,*Q*_*z*_) in Fig. 12b is reshaped into a matrix ***X***(*samples*, *features*), where each sample is a spatial position (samples = 700 × 700 pixels) with which is associated a feature vector, given by the diffracted intensity *I*(*Q*_*z*_) (features = 56 *Q*_*z*_ points). The non-negative matrix factorization of ***X*** into low-rank factors (***V***_***k***_**)** and sample distributions (***U***_***k***_) are shown in Fig. 12 (note that size(***X***) =  49,000 × 56 and * k * = 6 representatives). The low-rank factors ***V***_***k***_ can be readily interpreted as XRD scans associated with different structural “phases” in the SRO and PZT films, while their associated ***U***_***k***_ show the spatial configurations of such phases (note that each ***U***_***k***_ is reshaped from an *n* vector to an *x* × *y* image).

**Fig. 13:**
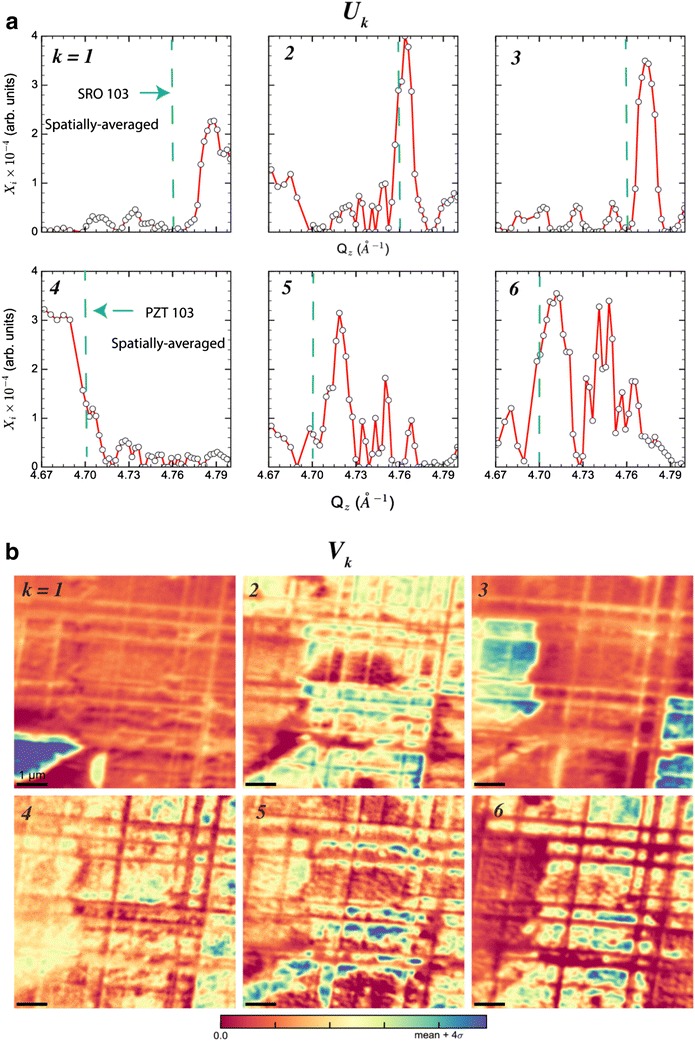
Non-negative matrix factorization of X-ray diffraction microscopy. **a** The low-rank factors and **b** the sample distributions resultant from applying NMF to the XDM dataset in Fig. 1b, with *k* = 6 representatives. The low-rank factors are readily interpreted as different classes of spatially resolved XRD scans, with *k* = 1–3 belonging to SRO and *k* = 4–6 to PZT. In each **U**_**k**_, a dashed line indicates the *Q*_*z*_ position of the SRO or PZT 103 Bragg peak as measured with standard XRD (i.e., spatially averaged across the entire sample). Note that each **U**_**k**_ is associated with a single **V**_**k**_, whereby the image intensity in **V**_**k**_ indicates the strength of association between sample regions and the structure encapsulated by the diffracted intensity in **U**_**k**_

Closer inspection of the low-rank factors indicates that *k * = 1–3 represent SRO domains with different *d*_103_ (where d_HKL_ is the spacing between (HKL) Bragg planes) as can be clearly seen from a shift in *Q*_*z*_ of their Bragg peak positions (Fig. 13a) with respect to the spatially averaged 103 reflection. The spatial distributions of SRO domains with different epitaxial strain states are given by their corresponding sample distributions (***U***_***k***_, *with k * = 1–3) as shown in Fig. 13b. Note that the intensity of each ***U***_***k***_ image is directly proportional to how strongly a particular region of the sample is associated with the structural state characterized by X-ray diffraction scan in ***V***_***k***_. In essence, NMF provides the spatial distributions of different classes of SRO lattice configuration (given by ***U***_***k***_), whose atomic positions, occupancies, etc. can be extracted through structural refinement of the XRD scan given by ***U***_***k***_.

The presence of SRO domains with different lattice constants is consistent with the broadening of the spatially averaged Bragg peak in (Fig. 12a), and a direct consequence of relieving the misfit strain imposed by the STO substrate. In addition, to a coherent relaxation of strain, with spatial variations in *d*_103_ that are localized around the misfit dislocation lines, as can be seen in ***V***_***2***_, there is a significant amount of incoherent strain relaxation leading to SRO domain segregation with no discernible preference to principal crystallographic directions (seen in ***V***_***1***_ and ***V***_***3***_). Such domain segregation in SRO could be associated with the presence of RuO_2_ precipitations [89], and can be directly checked through traditional structural refinement of (***U***_***1***_, ***V***_***1***_) and (***U***_***3***_, ***V***_***3***_) to obtain atomic occupancies of the unit cell in these different SRO domains, buried underneath the PZT layers. Similar to the structural states of SrRuO_3_, one can directly associate *k *=4–6 as containing structural deviations of PZT domains from the ensemble-averaged lattice configuration (*c *= 4.19 ± 10^−2^ Å, *a *= 3.97 ± 10^−2^ Å, as determined in [86]).

Without additional structural refinement, the NMF decomposition allows us to arrive at a qualitative understanding regarding the epitaxial strain transfer in this hetero-structure. For instance, note that by inspection of ***V***_***3***_ (SRO) and ***V***_***6***_ (PZT), we remark that SRO domains with lower than average *d*_103_ spacing induce a minor change in the *d*-spacing of PZT at the exact same lateral position. Furthermore, the changes in *d*-spacing of PZT as shown in ***V***_5,6_ is found to be largely concentrated near the misfit dislocations. These two observations indicate that strain transfer from one film to the next is mainly mediated by misfit dislocations of SRO which extend through PZT.

The power of matrix factorization techniques applied to structural imaging techniques such as XDM, resides in its ability to facilitate the extraction of key qualitative structural information, which can be additionally refined through model-based interpretations (e.g., crystal structure factor calculations). Additional applications of NMF and other matrix factorization techniques to other X-ray diffraction imaging techniques promise to reveal a wealth of structural information.

## Conclusion

In this tutorial paper, we discussed the utility of matrix factorization for performing linear unmixing of imaging and spectroscopic data commonly acquired via microscopy modalities. We presented a matrix factorization framework to implement different physical constraints such as sparsity, spatial smoothness, and non-negativity to constrain the unmixing, leading to more meaningful and interpretable endmembers and abundance maps. We compared the benefits of enforcing different physical constraints on ToF-SIMS data such as non-negativity (NMF), orthogonality without non-negativity (PCA), spatial smoothness, and sparsity on the resulting spectra and abundance maps. Finally, we presented detailed examples of the use of constrained matrix factorization approaches on different spectroscopy data, including X-ray microscopy and scanning probe microscopy datasets. This paper uses the open source NMF implementation from https://github.com/ramkikannan/nmflibrary. The imposition of such physical constraints here and in other machine-learning algorithms will be critical to better understand physical mechanisms in large multidimensional datasets commonly acquired in modern-day imaging facilities.
